# Predictors of Salvage After Re‐Exploration of Compromised Free Anterolateral Thigh Flaps for Post‐Traumatic Extremity Reconstruction

**DOI:** 10.1002/micr.70311

**Published:** 2026-10-22

**Authors:** Jannik Hinzmann, Suzan Kalkan, Sonja Verena Schmidt, Yonca Steubing, Marius Drysch, Flemming Puscz, Maxi von Glinski, Marcus Lehnhardt, Christoph Wallner

**Affiliations:** ^1^ Department of Plastic Surgery BG University Hospital Bergmannsheil Bochum, Ruhr University Bochum Bochum Germany

**Keywords:** extremity reconstruction, flap salvage, free flap failure, machine learning, Microsurgery, post‐traumatic soft‐tissue defects, risk factors

## Abstract

**Introduction:**

Vascular compromise after free anterolateral thigh (ALT) flap reconstruction is a time‐critical event and may result in total flap loss despite re‐exploration. Although earlier re‐exploration is associated with improved salvage, this relationship is often simplified by arbitrary thresholds, and internally validated prediction models remain scarce. We aimed to quantify this relationship and assess the additional predictive value of systemic factors beyond timing.

**Methods:**

This retrospective single‐center cohort study included all free ALT flaps for post‐traumatic extremity reconstruction between 2005 and 2020 that underwent re‐exploration for suspected vascular compromise on postoperative day (POD) 0–4. The primary endpoint was flap salvage at POD 5. Intraoperative findings were compared between salvaged and failed flaps. Univariable logistic regression examined the association between POD and salvage, followed by a multivariable model with prespecified covariates. Restricted cubic splines assessed nonlinearity, and 1000 bootstrap resamples were used to obtain optimism‐corrected estimates of discrimination and calibration.

**Results:**

Among 222 ALT flaps, 58 (26.1%) required re‐exploration; 33 (56.9%) were salvaged and 25 (43.1%) failed. Salvage rates were 90.0%, 51.7%, 37.5%, 57.1%, and 50.0% for re‐exploration on POD 0–4, respectively. Arterial and venous compromise were more frequent in failed flaps, without significant differences. Salvage probability crossed the 50% threshold between POD 1 and POD 2. Spline modeling demonstrated significant nonlinearity, with a steeper decline during the first two PODs followed by a relative plateau (LRT *p* = 0.037). Most systemic variables added little predictive information. Apparent discrimination was moderate (AUC 0.714), and optimism‐corrected performance was modest (AUC 0.622; calibration slope 0.69).

**Conclusion:**

Salvage probability declined in a biologically plausible, continuous, and nonlinear manner with increasing POD of re‐exploration, with a steeper decline at earlier time points, although salvage remained possible later. Timing appeared to be the principal determinant of salvage, while systemic variables added limited predictive value.

## 1 Introduction

Free tissue transfer is a highly reliable reconstructive technique, with contemporary series reporting overall flap survival rates exceeding 95% and total failure rates typically between 2% and 5% (Shen et al. 2021; Chen et al. 2007; Bui et al. 2007). Nevertheless, vascular compromise requiring urgent re‐exploration remains the most critical determinant of ultimate flap survival. Across large single‐center and systematic series, approximately 5%–10% of free flaps undergo take‐back for suspected pedicle compromise, with salvage rates among re‐explored flaps ranging from 50% to 80% (Shen et al. 2021; Chen et al. 2007; Bui et al. 2007; Bigdeli et al. 2019).

Most clinically relevant events occur within the first 24–72 postoperative hours (Shen et al. 2021; Chen et al. 2007; Bui et al. 2007). In a systematic review encompassing over 44,000 flaps, Shen et al. demonstrated that salvage success was highest when re‐exploration occurred on postoperative day (POD) 0–1 (93.8%), declined on POD 2 (83.3%), and fell sharply thereafter (Shen et al. 2021). Similarly, large institutional cohorts have shown that earlier re‐exploration after detection of compromise is strongly associated with higher salvage rates (Chen et al. 2007; Bui et al. 2007). Hour‐level analyses suggest that even short intra‐day delays reduce salvage probability (Bui et al. 2007; Kamali et al. 2021).

In addition to timing, flap‐intrinsic and etiologic factors influence outcome. Venous compromise is consistently associated with higher salvage rates than arterial thrombosis (Shen et al. 2021; Bui et al. 2007; Odorico et al. 2023), and fasciocutaneous flaps demonstrate greater salvageability than purely myocutaneous flaps (Odorico et al. 2023). Hypercoagulable states and prior take‐backs have also been linked to lower salvage success (Odorico et al. 2023; Carney et al. 2018). In contrast, systemic physiologic variables, such as hemoglobin level, transfusion, comorbidities, or ASA status, have shown inconsistent and often attenuated associations with salvage once timing and local factors are considered (Odorico et al. 2023; Carney et al. 2018; Lese et al. 2021). However, the incremental predictive value of systemic physiologic variables beyond timing and local factors remains unclear.

Despite broad agreement that “earlier is better”, the analytic treatment of timing in the salvage literature remains limited. Most studies dichotomize time to re‐exploration using arbitrary thresholds (e.g., ≤ 3 h vs. > 3 h or ≤ 72 h vs. > 72 h) (Kamali et al. 2021; Odorico et al. 2023), thereby imposing step functions on what is likely biologically a continuous ischemic process. Only rarely is time modeled as a continuous predictor, and formal evaluation of non‐linearity is uncommon. Furthermore, although several cohorts report multivariable analyses of salvage predictors, few provide discrimination metrics, calibration assessment, or internal validation. Bootstrap‐based optimism correction, a resampling method used to quantify and adjust for overoptimism in model performance when models are evaluated in the derivation dataset, is virtually absent from the salvage‐specific literature (Odorico et al. 2023; Carney et al. 2018). As a result, no internally validated prediction model currently provides individualized estimates of salvage probability conditioned on time to re‐exploration and clinically relevant covariates.

We therefore sought to quantify the relationship between timing of re‐exploration and flap salvage in a homogeneous cohort of free anterolateral thigh (ALT) flaps for post‐traumatic extremity reconstruction using time as a continuous predictor within a multivariable framework, and to assess the incremental value of systemic physiologic variables beyond timing and flap‐related factors. By applying bootstrap‐based internal validation and reporting optimism‐corrected measures of discrimination and calibration, our aim was to provide a methodologically robust estimate of salvage probability that reflects the continuous, time‐dependent nature of ischemic injury in microsurgical reconstruction.

## 2 Methods

### 2.1 Study Design and Population

This retrospective single‐center cohort study included all consecutive free ALT flaps used for reconstruction of post‐traumatic soft‐tissue defects of the limbs and performed between 2005 and 2020 that required surgical re‐exploration for suspected vascular compromise. Postoperative clinical flap monitoring was performed every 1–2 h through POD 5 and included assessment of capillary refill, turgor, and flap temperature, with the decision to re‐explore made by the on‐call surgeon. The primary outcome was flap salvage at POD 5, defined as preservation of more than 50% of the flap without compromising the intended reconstructive goal. Flap failure was defined as complete flap loss within this period. The timing of intervention was operationalized as the postoperative day of first surgical revision, recorded as an integer value (0–4). Intraoperative findings at first re‐exploration were grouped as arterial compromise, venous compromise, hematoma/bleeding, and other findings. Multiple findings could be recorded for the same flap.

### 2.2 Candidate Predictors

Candidate predictors were pre‐specified based on clinical reasoning and prior literature, comprising: (Shen et al. 2021) timing factors (POD of revision); (Chen et al. 2007) perfusion‐related parameters (postoperative hemoglobin, intraoperative hemoglobin loss, red blood cell units transfused postoperatively); (Bui et al. 2007) coagulation markers (Quick value, partial thromboplastin time, platelet count); (Bigdeli et al. 2019) operative factors (operative duration); and (Kamali et al. 2021) baseline patient characteristics (age, body mass index (BMI), American Society of Anesthesiologists physical status score). All continuous variables were analyzed on their original scales; no data‐driven variable selection procedures (e.g., stepwise selection) were employed to avoid overfitting and selection bias. Postoperative variables were defined as measurements recorded after the initial operation and prior to the revision procedure.

### 2.3 Statistical Analysis

The overall distributions of intraoperative findings were compared between salvaged and failed flaps using two‐sided Fisher's exact tests. POD‐stratified findings were summarized descriptively because of sparse cell counts. Univariable analysis of the association between timing of revision and salvage probability was performed using logistic regression, with POD modeled as a continuous linear predictor. Confidence intervals for the regression curve were estimated using bootstrap resampling with 1000 iterations.

For multivariable analysis, we constructed a logistic regression model including pre‐specified predictors. Given the limited sample size (58 events in total, with 33 salvaged and 25 failures), we restricted the final model to five predictors to maintain an events‐per‐variable ratio of approximately 5:1, consistent with recommendations for prediction modeling in small samples (van Smeden et al. 2019; Austin and Steyerberg 2017). The final predictors were selected from the candidate set based on their apparent predictive value and clinical relevance. Continuous predictors were standardized (mean = 0, SD = 1) prior to model fitting to enable comparison of effect sizes across variables with different units.

### 2.4 Internal Validation

Internal validation was performed using bootstrap resampling with 1000 iterations following established methods for optimism correction (Austin and Steyerberg 2017; Fahey et al. 2017). For each bootstrap sample, a model was fitted and its performance was evaluated on both the bootstrap sample (training performance) and the original dataset (test performance). The optimism was calculated as the difference between training and test performance, and the optimism‐corrected estimate was derived by subtracting the mean optimism from the apparent performance.

Discrimination was assessed using the area under the receiver operating characteristic curve (AUC). Calibration was evaluated graphically using calibration plots comparing predicted probabilities against observed proportions in quintile groups, and quantitatively using the calibration slope and intercept derived from logistic calibration regression. A calibration slope less than 1.0 indicates overfitting with regression to the mean expected in new samples.

The Brier score was calculated as the mean squared difference between predicted probabilities and observed binary outcomes, providing a combined measure of discrimination and calibration ranging from 0 (perfect) to 0.25 (non‐informative for balanced outcomes).

### 2.5 Missing Data

Patients with missing values for any predictor variable were excluded from the respective analyses (complete case analysis). The proportion of missing data for each variable was less than 5%.

### 2.6 Software

All analyses were performed using Python (version 3.12) with the following packages: pandas for data management, scikit‐learn (version 1.8) for logistic regression and performance metrics, statsmodels (version 0.14) for coefficient estimation with confidence intervals, and matplotlib for visualization. Bootstrap validation was implemented using custom functions with NumPy random sampling.

### 2.7 Reporting Standards

This study adheres to the Transparent Reporting of a multivariable prediction model for Individual Prognosis or Diagnosis (TRIPOD) guidelines for prediction model development.

This study was performed in accordance with the ethical standards of the Declaration of Helsinki.

## 3 Results

### 3.1 Cohort Characteristics

A total of 222 ALT‐flap transplantations for post‐traumatic extremity reconstruction were carried out between January 2005 and July 2020 in our institution. 58 ALT‐flaps required surgical re‐exploration for suspected vascular compromise within the first 5 postoperative days and met the inclusion criteria. Flap salvage was achieved in 33 cases (56.9%), whereas 25 flaps (43.1%) resulted in total loss. Of the salvaged flaps, 9 (27.3%) developed partial necrosis. Revision occurred on postoperative day (POD) 0 in 10 cases, POD 1 in 29 cases, POD 2 in 8 cases, POD 3 in 7 cases, and POD 4 in 4 cases.

### 3.2 Timing of Revision and Salvage Probability

Observed salvage rates were 90.0%, 51.7%, 37.5%, 57.1%, and 50.0% for re‐exploration on POD 0, 1, 2, 3, and 4, respectively (Figure 1). The fitted univariable logistic regression model showed a continuous decrease in odds of salvage per postoperative day. The 95% bootstrap confidence band indicated increasing uncertainty at later PODs due to smaller group sizes. The fitted curve crossed the 50% predicted salvage probability between POD 1 and POD 2. These findings support a time‐dependent decay in salvage probability rather than a discrete early/late threshold.

**FIGURE 1:**
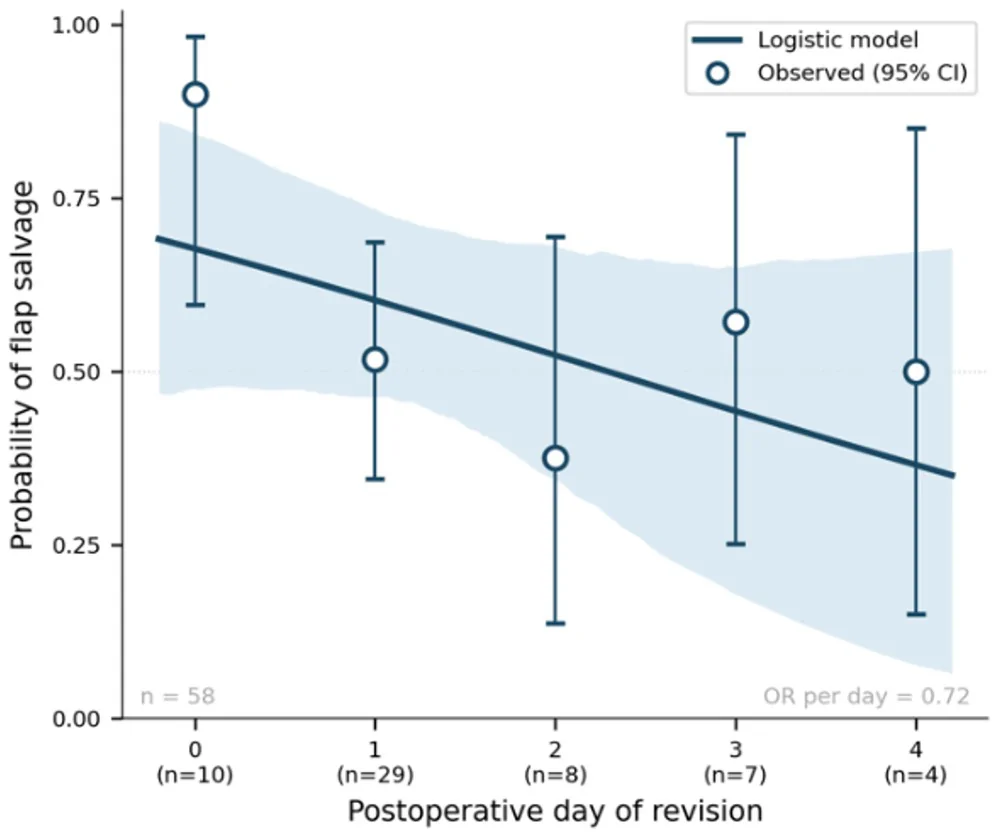
Probability of flap salvage as a function of timing of surgical revision. Salvage probability following microsurgical flap revision is shown as a function of postoperative day (POD) of first re‐exploration. Open circles represent observed salvage proportions at each POD with 95% Wilson score confidence intervals; sample sizes per group are indicated on the *x*‐axis (POD 0: *N* = 10; POD 1: *N* = 29; POD 2: *N* = 8; POD 3: *N* = 7; POD 4: *N* = 4). The solid line represents the fitted univariable logistic regression model; the shaded band indicates the 95% bootstrap confidence interval based on 1000 resamples. The horizontal dotted line marks the 50% salvage probability threshold. OR: odds ratio; POD: postoperative day; CI: confidence interval.

### 3.3 Intraoperative Findings at First Re‐Exploration

Numerically, arterial compromise (40.0% vs. 18.2%) and venous compromise (52.0% vs. 36.4%) were more frequent among failed than salvaged flaps, whereas hematoma/bleeding was less frequent among failed flaps (16.0% vs. 36.4%). None of the overall differences reached statistical significance (Table 1).

**TABLE 1:** Intraoperative findings at first re‐exploration by early flap outcome and postoperative day.

Overall distribution by early flap outcome			
Intraoperative finding	Salvaged flaps (*n* = 33)	Failed flaps (*n* = 25)	*p*
Arterial compromise	6 (18.2)	10 (40.0)	0.081
Venous compromise	12 (36.4)	13 (52.0)	0.289
Hematoma/bleeding	12 (36.4)	4 (16.0)	0.138
Other findings	5 (15.2)	7 (28.0)	0.329

Distribution by postoperative day					
Intraoperative finding	POD 0, S/F (9/1)	POD 1, S/F (15/14)	POD 2, S/F (3/5)	POD 3, S/F (4/3)	POD 4, S/F (2/2)
Arterial compromise	0/1	2/8	3/0	1/1	0/0
Venous compromise	2/0	4/7	2/3	2/2	2/1
Hematoma/bleeding	4/0	6/1	0/2	1/1	1/0
Other findings	3/0	2/2	0/3	0/1	0/1

*Note:* Data in the first section are given as *n* (% within outcome group). *p* values were calculated using two‐sided Fisher's exact tests. Data in the second section are presented as the number of salvaged/failed flaps with the respective intraoperative finding and are descriptive only. Multiple findings could be recorded for the same flap and categories were therefore not mutually exclusive. Other findings included acute infection, pedicle torsion, and other rare findings.

Abbreviations: POD: postoperative day; S/F: salvaged/failed.

### 3.4 Perioperative Physiologic Trajectories

Temporal trajectories of hemoglobin, transfusion requirements, platelet count, and Quick value stratified by outcome are shown in Figure 2. Although individual variability was substantial, no clear or consistent separation between salvaged and failed flaps was observed across POD 0–4 for hemoglobin concentration, platelet count, or coagulation parameters. Red blood cell transfusion showed slightly higher mean values in failed flaps. However, overlap between groups was considerable. Overall, systemic physiologic variables demonstrated limited discriminatory capacity when considered descriptively over time.

**FIGURE 2:**
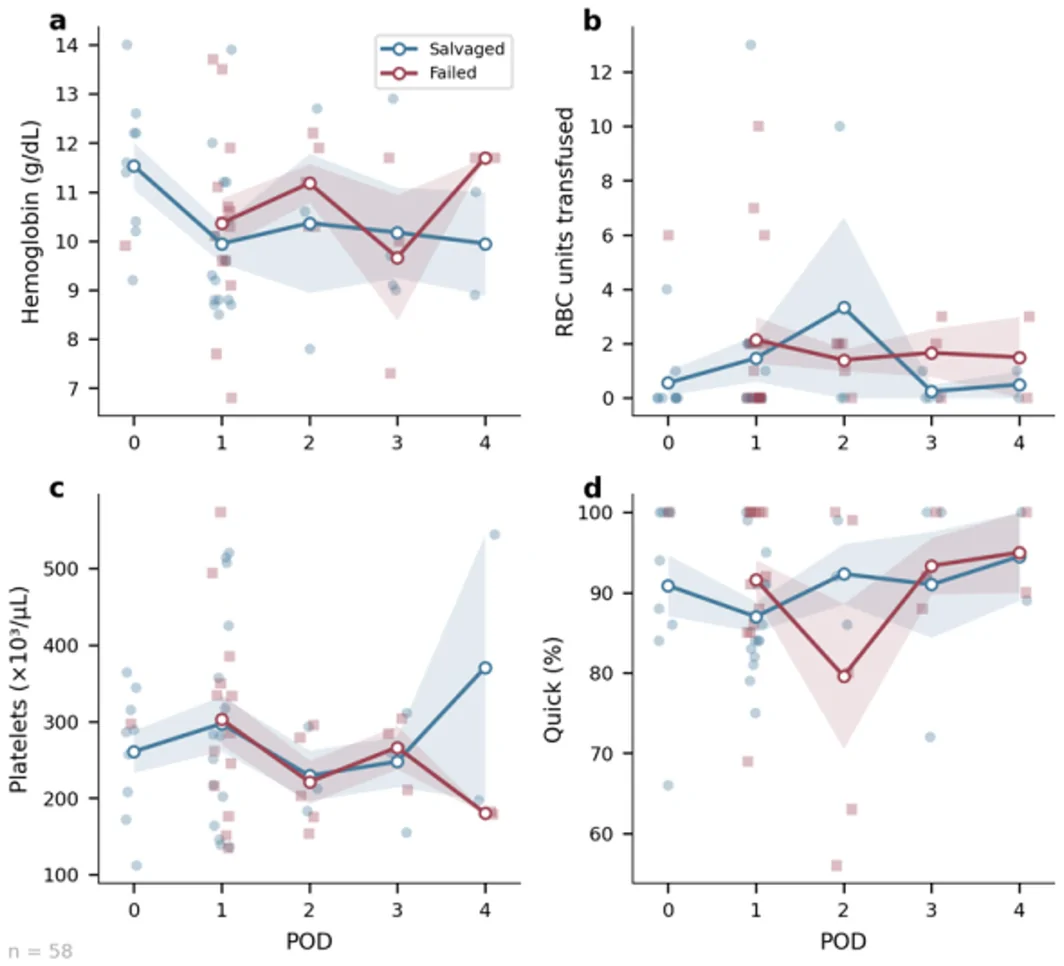
Perioperative physiologic trends stratified by flap outcome. Temporal trajectories of four physiologic parameters are compared between salvaged (*n* = 33) and failed (*n* = 25) free flaps across postoperative days 0–4. Panel (a) postoperative hemoglobin concentration. Panel (b) red blood cell units transfused. Panel (c) platelet count. Panel (d) Quick coagulation value. Individual observations are shown as scatter points; lines connect group means at each POD with shaded bands representing ±1 standard error of the mean. POD: postoperative day; RBC: red blood cell.

### 3.5 Multivariable Model and Internal Validation

A multivariable logistic regression model including five pre‐specified predictors (POD of revision, postoperative hemoglobin, BMI, age, ASA status) was constructed. The apparent discrimination of the model was moderate (AUC = 0.714). After bootstrap‐based optimism correction (1000 resamples), discrimination decreased to an AUC of 0.622 (Figure 3). The apparent Brier score of 0.214 increased to 0.256 after optimism correction, indicating reduced predictive accuracy in resampled datasets.

**FIGURE 3:**
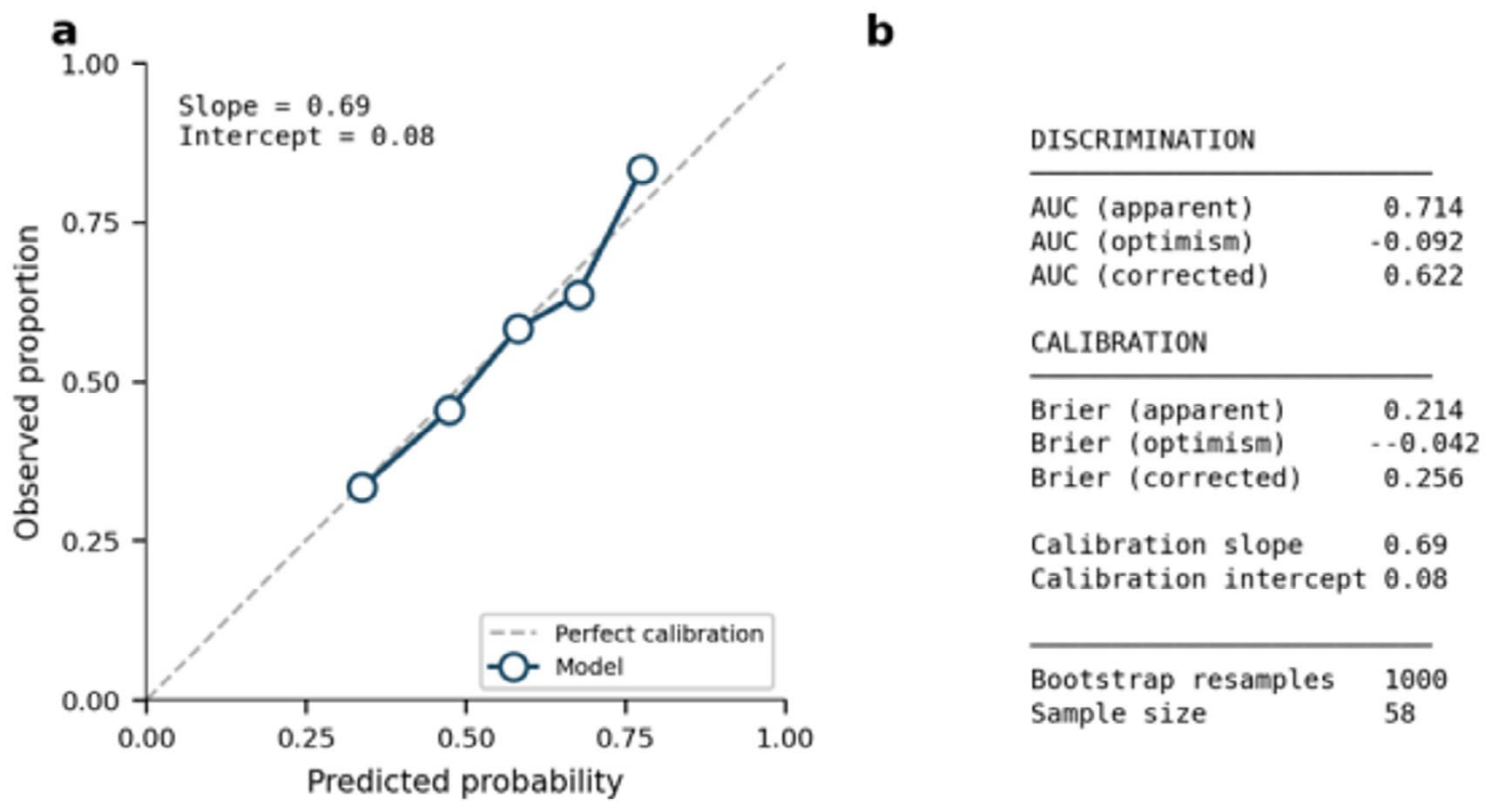
Internal validation of the multivariable prediction model. Panel (a) displays the calibration plot comparing predicted salvage probabilities against observed proportions in quintile‐based probability groups. The dashed diagonal line represents perfect calibration; the solid line with markers shows model performance. Calibration slope and intercept were derived from bootstrap validation. Panel (b) summarizes discrimination and calibration metrics. All performance estimates are optimism‐corrected using bootstrap resampling with 1000 iterations. The apparent area under the receiver operating characteristic curve (AUC) was 0.714 after optimism correction; the AUC was 0.622. The Brier score increased from 0.214 (apparent) to 0.256 (optimism‐corrected), indicating expected performance degradation in new samples. The calibration slope of 0.69 suggests moderate overfitting. The model included five pre‐specified predictors (POD of revision, postoperative hemoglobin, body mass index, age, ASA score) in 58 flaps with complete data. AUC: area under the curve; POD: postoperative day; ASA: American Society of Anesthesiologists physical status score.

Calibration analysis demonstrated a calibration slope of 0.69, consistent with moderate overfitting and regression toward the mean in new samples (Figure 3a). Thus, while the model retained modest discriminatory ability after correction, performance metrics indicate limited robustness in this small cohort.

### 3.6 Independent Predictors of Salvage

Standardized odds ratios from the multivariable model are displayed in Figure 4. Timing of revision showed a negative association with salvage, consistent with the univariable analysis, although confidence intervals were wide. Among baseline characteristics, BMI emerged as the only statistically significant predictor (OR 2.06, 95% CI 1.04–4.08). Age, ASA score, and postoperative hemoglobin did not demonstrate independent associations with salvage. Overall, effect sizes for systemic physiologic parameters were small and statistically non‐significant, suggesting limited incremental predictive value beyond timing.

**FIGURE 4:**
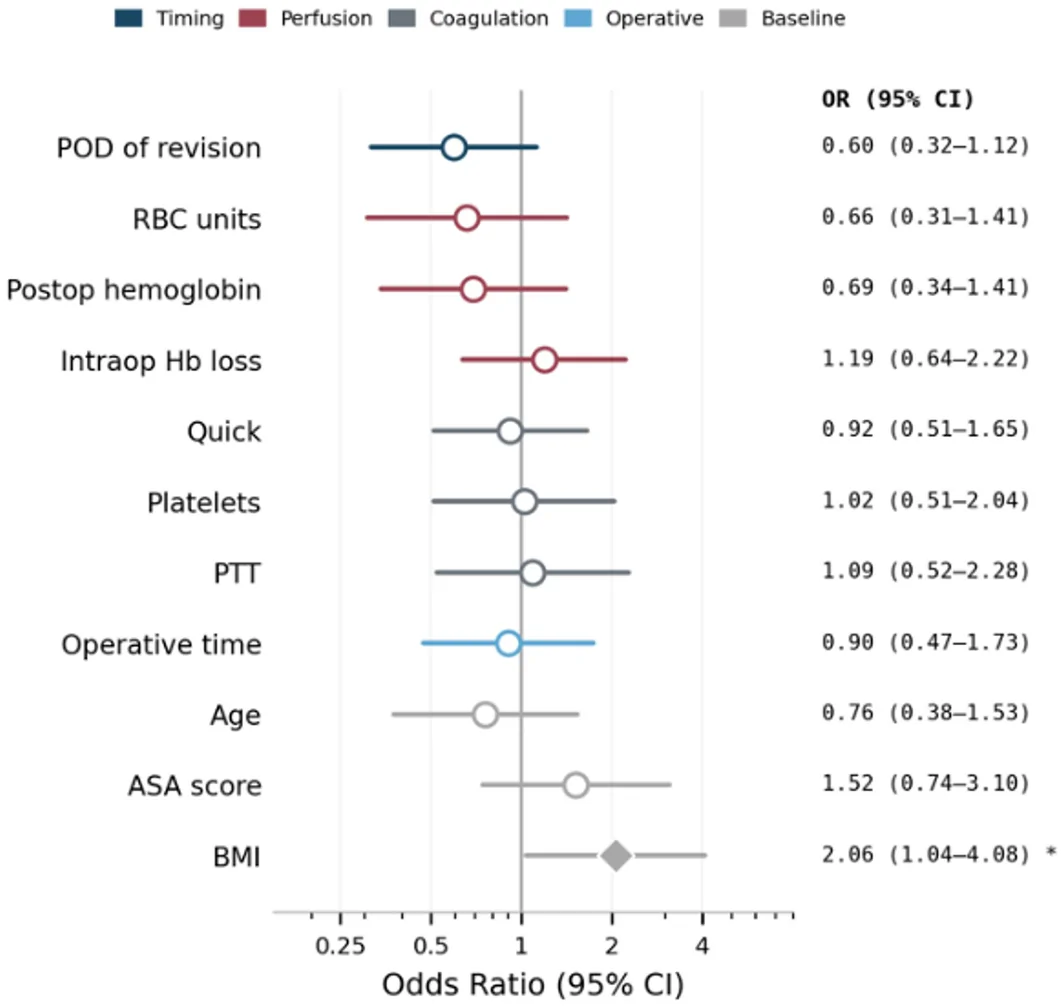
Independent determinants of flap salvage: Multivariable logistic regression. Forest plot displaying odds ratios (OR) with 95% confidence intervals for standardized predictors (per 1 standard deviation change) of flap salvage. Predictors are grouped by conceptual domain: Timing (blue), Perfusion (red), Coagulation (gray), Operative factors (light blue), and Baseline characteristics (light gray). Diamond markers indicate statistically significant predictors (*p* < 0.05); open circles indicate non‐significant associations. Body mass index was the only predictor reaching statistical significance (OR 2.06, 95% CI 1.04–4.08). The x‐axis is displayed on a logarithmic scale with a vertical reference line at OR = 1.0 (no effect). Model performance metrics are optimism‐corrected via bootstrap resampling (*n* = 1000): AUC = 0.622, Brier score = 0.256. OR: odds ratio; CI: confidence interval; POD: postoperative day; Hb: hemoglobin; PTT: partial thromboplastin time; BMI: body mass index; ASA: American Society of Anesthesiologists physical status classification; AUC: Area under the receiver operating characteristic curve.

### 3.7 Nonlinear Association Between Timing of Revision and Flap Salvage

Time to re‐exploration was significantly associated with flap salvage probability in the primary logistic regression model, with an odds ratio of 0.72 per additional postoperative day. To evaluate the assumption of linearity, timing was additionally modeled using restricted cubic splines with three knots (Figure 5). A likelihood ratio test demonstrated significant nonlinearity (*χ* ^2^ = 4.34, *p* = 0.037). Model fit improved with the spline specification compared to the linear model (AIC 79.1 vs. 81.5).

**FIGURE 5:**
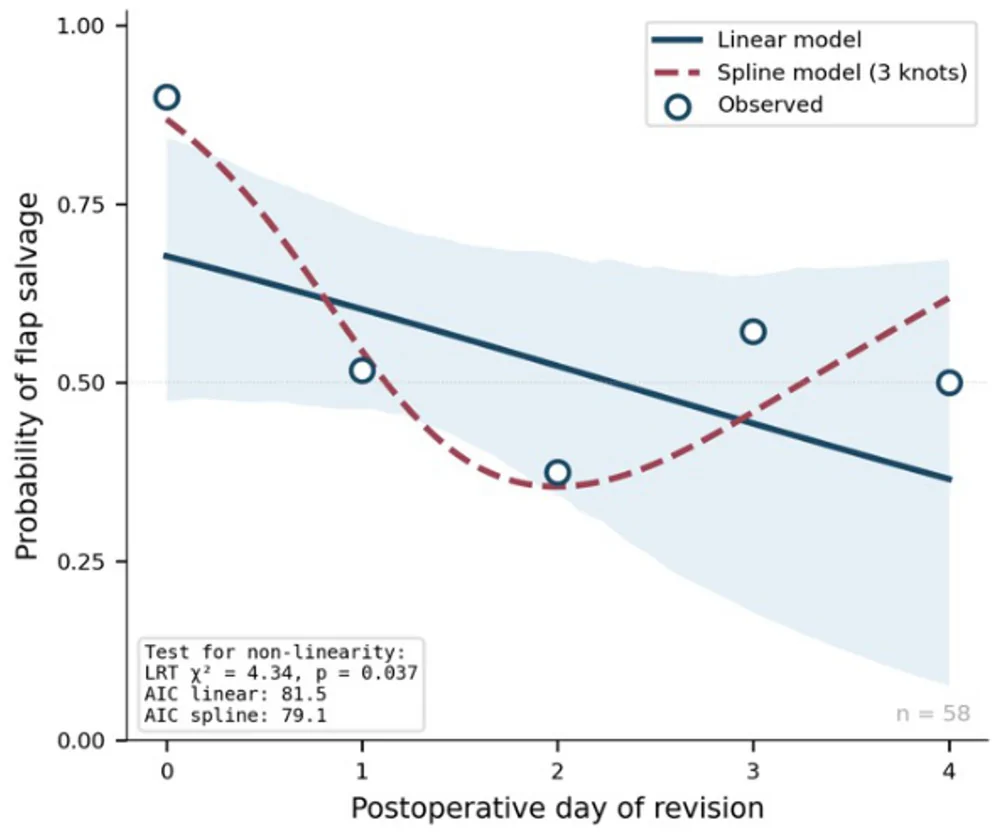
Assessment of non‐linearity in the timing‐salvage relationship. Predicted salvage probability as a function of POD of revision, comparing a linear logistic regression model (solid blue line with 95% bootstrap confidence band) versus a restricted cubic spline model with 3 knots (dashed red line). Open circles represent observed salvage proportions at each POD. Non‐linearity was tested using a likelihood ratio test comparing nested models. The spline model showed marginally better fit (AIC 79.1 vs. 81.5; LRT χ^2^ = 4.34, *p* = 0.037), suggesting the decline in salvage probability may be steeper in early postoperative days. Confidence intervals are based on 1000 bootstrap resamples. POD, postoperative day; AIC, Akaike information criterion; LRT, likelihood ratio test.

Graphically, the spline model demonstrated a steep decline in salvage probability during the first two postoperative days, followed by a relative plateau at later time points. These findings suggest that the effect of delay is not constant across time but follows a nonlinear decay pattern, with the most pronounced reduction in salvage probability in the first days.

### 3.8 Decision Curve Analysis

Decision curve analysis was performed using optimism‐corrected estimates derived from 1000 bootstrap resamples (Figure 6). The model demonstrated a positive net benefit compared with both “treat‐all” and “treat‐none” strategies across threshold probabilities ranging from 0.23 to 0.80. The greatest separation from the treat‐all strategy was observed within the intermediate threshold range (approximately 0.30–0.60), while confidence bands widened at higher thresholds.

**FIGURE 6:**
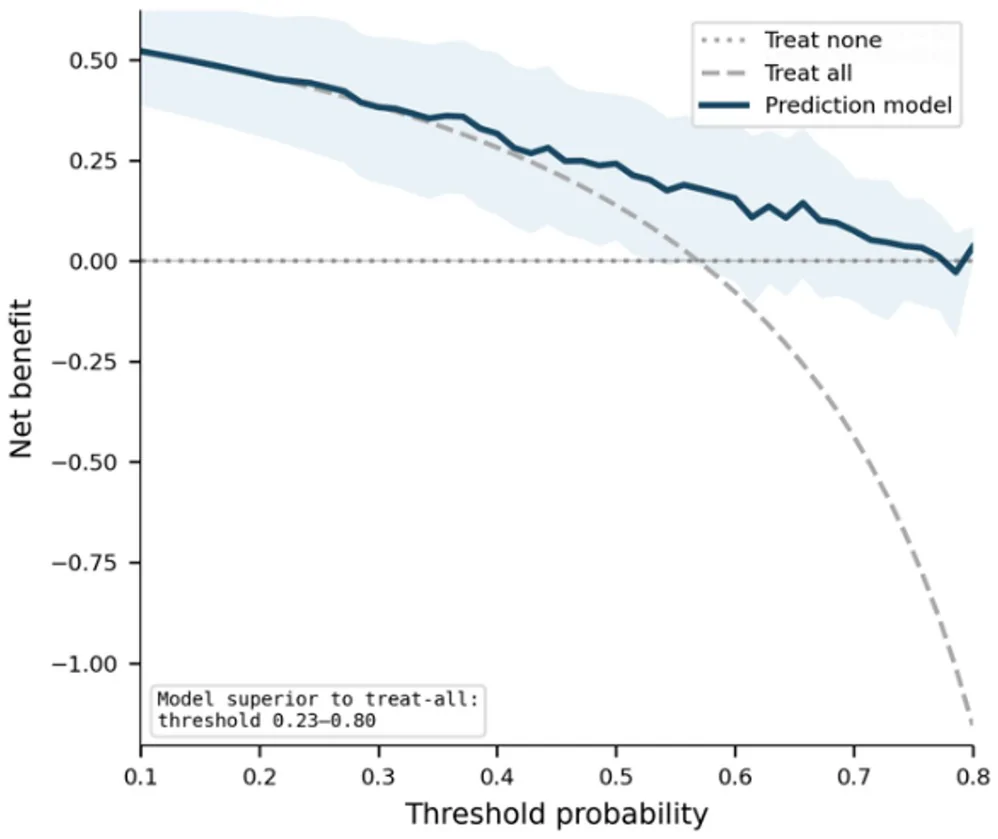
Decision curve analysis for the multivariable prediction model. Net benefit plotted against threshold probability for the 5‐variable prediction model (solid blue line with 95% bootstrap confidence band), compared with treat‐all (dashed gray line) and treat‐none (dotted gray line) strategies. Net benefit is defined as the proportion of true positives minus the proportion of false positives weighted by the odds of the threshold probability. Predictions were optimism‐corrected using bootstrap resampling (*n* = 1000). The model provides incremental clinical utility over the treat‐all strategy for threshold probabilities between 0.23 and 0.80. Given the moderate discrimination (optimism‐corrected AUC = 0.622) and small sample size (*n* = 58), these findings should be interpreted as exploratory. Net benefit values below zero indicate harm from model‐guided decisions at that threshold. AUC, Area under the receiver operating characteristic curve.

## 4 Discussion

Reconstruction of soft‐tissue defects following severe trauma of the upper or lower extremity remains a challenging task which often requires sophisticated coverage through free flap transplantation. Although reported survival rates for free flaps exceed 95% in the literature (Shen et al. 2021; Chen et al. 2007; Bui et al. 2007; Zhang et al. 2019; Xiong et al. 2016), vascular compromise may occur, requiring urgent re‐exploration and possibly ending in total flap loss. In this single‐center cohort of compromised free ALT flaps after traumatic extremity reconstruction, salvage probability declined with increasing POD of re‐exploration and crossed the 50% predicted salvage probability between POD 1 and POD 2, whereas systemic physiologic variables contributed little additional predictive information. However, the association between salvage and timing was not completely linear, as spline modeling suggested a steeper decline in salvage probability during the first two PODs followed by a relative plateau later. This temporal pattern supports the concept of a continuous, time‐dependent ischemic injury process rather than a discrete early/late threshold effect and suggests that arbitrary temporal cutoffs may therefore oversimplify this relationship. It is also worth mentioning that, contrary to the findings from other studies (Shen et al. 2021), successful revisions were achieved at POD 3 or POD 4, supporting continued flap monitoring through POD 5. A later POD should therefore not preclude re‐exploration when clinical findings indicate vascular compromise.

Our findings are directionally consistent with prior studies showing improved salvage with earlier take‐back (Shen et al. 2021; Chen et al. 2007; Bui et al. 2007), but extend the literature by treating timing as a continuous predictor and formally testing for nonlinearity rather than relying on arbitrary dichotomous cutoffs. However, direct comparison of absolute salvage and re‐exploration rates across studies is difficult due to substantial heterogeneity in free flap types, indications, monitoring protocols, definitions of compromise, and time metrics. The comparatively high re‐exploration rate in our cohort reflects the specific characteristics of this patient population. Unlike cohorts dominated by elective indications, our study focused exclusively on post‐traumatic extremity reconstruction. Patients requiring revision had undergone a mean of five prior operations at the recipient site. More than half were active smokers, over 15% had peripheral arterial disease, and comorbidities such as obesity and diabetes mellitus were frequent. Moreover, approximately half had a history of prior bone or soft‐tissue infection in the operative field. These features likely contributed to the elevated revision rate. The present study therefore offers greater internal consistency within a narrowly defined subgroup, while limiting generalizability to other reconstructive populations.

Although prior studies have reported higher salvage rates after venous than arterial compromise (Bui et al. 2007; Odorico et al. 2023), the small and overlapping groups in our cohort precluded firm conclusions regarding individual intraoperative findings. Beyond these local factors, another important finding is the limited incremental predictive value of systemic perioperative variables in this setting. The perioperative trajectories of hemoglobin, platelet count, and Quick value showed considerable overlap between salvaged and failed flaps between POD 0–4, and no consistent visual separation. The need for postoperative red blood cell transfusions tended to be higher in failed flaps, but the overlap remained considerable. In the multivariable model, age, ASA status, and postoperative hemoglobin showed no independent associations with salvage, while timing maintained a negative association. Taken together, these findings support the hypothesis that, naturally, once compromise has occurred, timing contributes more to the outcome of salvage than general systemic physiological parameters, at least as captured by routine perioperative measurements in a retrospective dataset. However, this does not exclude the possible influence of these factors on the development of flap compromise itself. A recent meta‐analysis from Claresta et al. failed to identify any factors that significantly increased the risk for flap loss though (Claresta Adhya Sastri et al. 2023). Among the covariates included, BMI was the only statistically significant independent predictor (standardized OR 2.06, 95% CI 1.04–4.08). This finding should be interpreted with caution. While it may reflect genuine differences in patient characteristics related to flap rescueability, it may also be due to residual confounding, selection effects, or sampling‐related signature amplified by the small cohort size. In particular, the direction and mechanism of this association are unclear and should not be overinterpreted without replication. This cautious interpretation is further supported by the literature, in which increased BMI/obesity has not generally been identified as an independent risk factor for total flap loss, although it does increase the overall risk of postoperative complications (Heidekrueger et al. 2021; Crippen et al. 2018; Stanton et al. 2024).

A major strength of this study is the explicit evaluation of model performance and internal validity. The apparent performance of the multivariable model was moderate (AUC 0.714; Brier score 0.214), but bootstrap‐based optimism correction substantially reduced discrimination (AUC 0.622) and worsened the Brier score (0.256), indicating that model performance was overestimated in the derivation sample. In addition, the calibration slope of 0.69 suggests moderate overfitting and implies that the apparent predictor effects (i.e., regression coefficients and corresponding odds ratios) were likely too extreme and would be expected to attenuate in new data. These findings are important because many salvage studies report multivariable associations without calibration assessment or internal validation (Odorico et al. 2023; Carney et al. 2018). The discrepancy between apparent and optimism‐corrected performance transparently demonstrates the degree of overoptimism inherent in this small salvage cohort and underscores the need for external validation before clinical application.

The decision curve analysis indicated a potential net benefit of the multivariable model compared to the “treat all” and “treat none” strategies across a wide range of threshold probabilities (approximately 0.23–0.80). By complementing optimism‐corrected discrimination and calibration with decision curve analysis, the present study evaluates not only statistical model performance but also its potential clinical usefulness. However, given the modest optimism‐corrected discrimination, limited sample size, and lack of external validation, these findings should be considered exploratory. The model should therefore be regarded as a tool for risk estimation rather than a clinical decision rule and should neither delay nor replace urgent re‐exploration when clinically indicated. External validation is required before any threshold‐based application in clinical practice.

This study has several limitations. Its retrospective, single‐center design is susceptible to selection and information biases, and the sample size limited the complexity and precision of the model. The long inclusion period may have introduced heterogeneity due to changes in surgical decision‐making and perioperative management. The timing was recorded as the POD of re‐exploration rather than as a precise time interval, limiting temporal granularity and potentially attenuating time‐dependent effects. Finally, the binary outcome focused on early flap status at POD 5 and therefore did not capture the full spectrum of postoperative morbidity. Limited partial necrosis that did not compromise the intended reconstructive goal was included within the salvage group rather than analyzed separately, while later partial necrosis, wound‐healing complications, and secondary procedures were not assessed.

## 5 Conclusion

Overall, our findings support a biologically plausible, continuous and nonlinear relationship between the timing of re‐exploration and flap salvage. In contrast, most routinely available perioperative clinical and laboratory factors showed no consistent independent association with salvage in this cohort. These findings underscore that suspected vascular compromise should be treated as a time‐critical event, while also indicating that salvage may remain possible at later time points. Larger, externally validated models with more granular timing data are needed for clinically useful individualized prediction.

## Author Contributions

**Jannik Hinzmann:** conceptualization; methodology; formal analysis; data curation; investigation; writing – original draft. **Suzan Kalkan:** investigation. **Sonja Verena Schmidt:** methodology; investigation; writing – review and editing. **Yonca Steubing:** investigation; writing – review and editing. **Marius Drysch:** writing – review and editing. **Flemming Puscz:** writing – review and editing. **Maxi von Glinski:** conceptualization; methodology; writing – review and editing. **Marcus Lehnhardt:** conceptualization; supervision; writing – review and editing. **Christoph Wallner:** conceptualization; methodology; formal analysis; data curation; supervision; writing – review and editing. All authors reviewed and approved the final version of the manuscript.

## Funding

The authors have nothing to report.

## Disclosure

A generative AI tool was used exclusively for language polishing and grammatical revision. All scientific content, analyses, interpretations, and final wording were reviewed and approved by the authors.

## Ethics Statement

Formal ethics committee approval was not required for this retrospective, non‐interventional analysis of anonymized clinical data in accordance with institutional requirements. Written informed consent for the processing and scientific use of anonymized clinical data was obtained from all patients as part of the preoperative consent process.

## Consent

Informed and written consent was obtained from the patients before the surgery. Informed consent involved the collection of their clinical data and biological material for use in biomedical research and further medical publishing.

## Conflicts of Interest

The authors declare no conflicts of interest.

## Data Availability

The data that support the findings of this study are available from the corresponding author upon reasonable request, subject to institutional and data protection requirements. The data are not publicly available due to patient privacy restrictions.
